# Is primary sarcopenia more common in patients with restless legs syndrome?

**DOI:** 10.1097/MD.0000000000049702

**Published:** 2026-07-17

**Authors:** Aysegul Akkan Suzan, Seyda Bilgin, Veysel Suzan

**Affiliations:** a Cerrahpasa Medical Faculty, Department of Neurology, Division of Clinical Neurophysiology, Istanbul University-Cerrahpasa, Istanbul, Turkey; b Cerrahpasa Medical Faculty, Department of Internal Medicine, Division of Geriatric Medicine, Istanbul University-Cerrahpasa, Istanbul, Turkey; c Department of Internal Medicine, Division of Geriatric Medicine, Istanbul Training and Research Hospital, Istanbul, Turkey.

**Keywords:** geriatric, inflammation, restless legs syndrome, sarcopenia

## Abstract

This study aimed to investigate the frequency of primary sarcopenia in individuals with and without restless legs syndrome (RLS). In this cross-sectional study, 114 patients who presented to our outpatient clinic for the first time and were diagnosed with RLS and 147 patients without RLS were consecutively included. Patients with malignancy, uncontrolled diabetes mellitus, or diseases that may cause secondary sarcopenia or neuropathy were excluded from the study. The European Working Group on Sarcopenia in Older People 2 criteria for the diagnosis of sarcopenia and the International RLS Study Group criteria for the diagnosis of RLS were applied. The association between RLS and sarcopenia was analyzed using a logistic regression analysis. There were no significant differences in terms of chronic diseases. While there was no significant difference between the groups with and without RLS (70.11 ± 9.56 vs 71.76 ± 9.61) in terms of age (*P* = .170), females were significantly higher in the RLS group (*P* = .001). The rate of sarcopenia was significantly higher in patients with RLS (54% vs 37%, *P* = .005). In the multivariate logistic regression analysis of sarcopenia and sex (female), it was determined that both were associated with RLS [odds ratio 1.941 (95%) confidence interval 1.107–3.402, *P* = .021; odds ratio 2.733, (95%) confidence interval 1.442–5.177, *P* = .002], respectively). Primary sarcopenia is more common in patients with RLS than in those without RLS. Screening for sarcopenia should be considered in patients with restless leg syndrome.

## 1. Introduction

The third decade is accepted as the milestone for skeletal muscle mass, with age 27 being the threshold at which muscle mass begins to be negatively associated with age in both men and women.^[1]^ The definition of sarcopenia by the European Working Group on Sarcopenia in Older People (EWGSOP) was updated in 2010 and 2019 (EWGSOP2). In accordance with EWGSOP2, muscle function (strength) should be assessed first for the diagnosis of sarcopenia, which is considered “probable” when low muscle strength is detected. “Sarcopenia” is diagnosed if “low muscle mass is also present in a person with low muscle strength. The coexistence of decreased muscle strength, muscle mass, and physical performance is referred as “severe sarcopenia.”^[2]^ Several factors have been proposed in the pathogenesis of sarcopenia, including changes in the systemic factors of muscle activity and differentiation, inflammation, oxidative stress, neuromuscular junction dysfunction, decreased numbers of motor units, insulin resistance, and mitochondrial dysfunctions.^[3–8]^ All these pathophysiological processes result in size and number declines in type II muscle fibers, intramuscular and intermuscular fat infiltration, and a decreased number of satellite cells, which are the main group of cells responsible for the replacement and repair of damaged muscle fibers.^[3]^ Sarcopenia is an important marker that plays a key role in the development of frailty in the elderly and has also been addressed within the broader spectrum of age-related conditions.^[9,10]^

Restless legs syndrome (RLS), also known as Willis-Ekbom disease, is a sensorimotor disorder characterized by an urgent need to move the legs, often accompanied by unpleasant sensations that worsen during rest or inactivity, particularly in the evening or at night, and is relieved by movement. These symptoms are distinct and not attributable to other medical conditions.^[11]^ Individuals with RLS have difficulty identifying symptoms. Patients usually describe these symptoms as an unavoidable desire to move their legs, tingling, a feeling of electric current, not very painful but uncomfortable.^[12]^ The pathophysiology of RLS remains unclear; however, brain iron deficiency and genetic and environmental predispositions are thought to be key factors.^[13]^ Iron acts as a cofactor for tyrosine hydroxylase, an enzyme that converts L-tyrosine to L-DOPA, highlighting its role in dopamine synthesis. Neuroimaging studies have emphasized the involvement of sensorimotor transmissions passing through the thalamus, including the mesolimbic and nigrostriatal dopaminergic pathways, opioid system, GABAergic, and glutamatergic transmission.^[14]^ A recent gene-environment interaction model suggests that genetic factors contribute to early onset, family history, and slow disease progression, whereas environmental factors, such as comorbid conditions, drug intake, and lifestyle habits, are associated with late-onset, sporadic cases, and rapid disease progression.^[13]^

RLS is a common neurological disorder that is particularly prevalent in older adults. RLS studies in geriatric populations report that the prevalence of RLS ranges between approximately 10% and 20%.^[15,16]^ The prevalence of sarcopenia varies significantly depending on the diagnostic criteria and characteristics of the population studied. Global meta-analyses typically estimate prevalence rates to be approximately 10% to 20%; however, notably higher rates have been observed in high-risk or clinical populations.^[17,18]^

While a direct mechanistic link between RLS and sarcopenia has not been explicitly examined in the extant literature, certain shared pathophysiological pathways suggest a biological intersection between these conditions. It is posited that the sleep disorder characteristic of RLS may adversely impact skeletal muscle repair processes by disrupting circadian rhythms. Additionally, chronic low-grade inflammation may exacerbate muscle catabolism through the action of pro-inflammatory cytokines, and the physical inactivity associated with RLS may further accelerate the loss of muscle mass. The presence of these common pathways provides a compelling rationale for investigating the relationship between these 2 conditions through prospective studies.

Building upon this rationale, the current study sought to examine the prevalence of primary sarcopenia among patients with and without RLS. To our knowledge, this represents the first prospective investigation of this association, with secondary causes of sarcopenia explicitly excluded to ensure the specificity of the results.

## 2. Materials and methods

In this cross-sectional study, patients over 60 years of age who were admitted to our geriatric outpatient clinic for the first time between December 1, 2023, and July 1, 2024, were included consecutively. They were evaluated simultaneously by both a geriatrics and a neurologist. The geriatric specialist assessed for sarcopenia. Patients diagnosed with a disease that might lead to secondary sarcopenia (malignancy, rheumatological disease, advanced organ failure, human immunodeficiency virus infection, inflammatory bowel diseases, malabsorption, steroid use, thyroid dysfunction, etc) were excluded from the study. Additionally, patients with uncontrolled diabetes mellitus in the last 6 months, untreated hypothyroidism or hyperthyroidism, and acute infections were excluded. The neurologist evaluated for RLS. Systemic diseases that could cause peripheral neuropathy and creatinine kinase levels above 150 units/liter (due to possible muscle disease) were excluded from the study. Patients in both groups were evaluated by the same specialists in the same outpatient clinic during the same time period. A total of 412 individuals were initially screened during the study period. Among them, 151 participants were excluded for the following reasons: malignancy (n = 34), rheumatologic disease (n = 12), advanced organ failure (n = 11), steroid use (n = 8), thyroid dysfunction (n = 8), malabsorption or inflammatory bowel disease (n = 7), uncontrolled diabetes mellitus (n = 14), acute infection (n = 15), possible peripheral neuropathy or elevated creatinine kinase levels (n = 14), inability to undergo bioelectrical impedance analysis (n = 20), and refusal to participate (n = 8). Consequently, 261 participants were included in the final analysis, including 114 patients with RLS and 147 without RLS.

The consensus criteria of EWGSOP2 for sarcopenia and the International RLS Study Group (IRLSSG) for RLS were applied for diagnosis.^[2,11]^ The SARC-F questionnaire was administered to patients according to the EWGSOP2 criteria.^[2]^ Handgrip strength (HGS) for muscle strength assessment, bioelectrical impedance analysis for muscle mass measurement, and the 6-m walking test for muscle function evaluation were used. A hand dynamometer (Takei TKK 5401 model; Takei Scientific Instruments Co., Tokyo, Japan) was used to assess grip strength. The test was performed with participants seated comfortably in a chair with their elbows at 90 °of flexion and their arms parallel to the floor. Each hand was tested 3 times with a 1-min resting periods, and the best effort value was recorded for all patients. In accordance with EWGSOP2 criteria, low HGS thresholds were also determined (for women <16 kg and for men <27 kg).^[2]^ The skeletal muscle index was calculated as appendicular skeletal muscle mass (ASM) divided by height squared (kg/m^2^), using a BIA device (Tanita Body Composition Analyzer® TBF-300 model; Tanita Co., Tokyo, Japan)) after a 12-hour fast^2^. Low muscle mass was defined according to EWGSOP2 cutoffs (<7.0 kg/m^2^ for men, <5.5 kg/m^2^ for women).^[2]^ The 6-m walking test was performed to assess physical activity performance. Using a chronometer, the walking speed of each patient was determined in m/s and recorded. A test result of ˃0.8 m/s was recognized as normal.^[2]^ As recommended by the EWGSOP2 criteria, patients with low HGS and skeletal muscle index were included in the sarcopenia group.^[2]^ The patients’ demographic data, chronic diseases, number of drugs used, and laboratory values (hemoglobin, ferritin, creatinine, thyroid-stimulating hormone, vitamin B12, 25-hydroxy vitamin-D, and hemoglobin A1c levels) were noted.

RLS was diagnosed based on the diagnostic criteria updated in 2014 by the IRLSSG. According to these criteria, all 5 essential conditions must be met to establish a diagnosis: an urge to move the legs, usually accompanied by uncomfortable or unpleasant sensations; symptoms that begin or worsen during periods of rest or inactivity; partial or complete relief of symptoms by movement; symptoms that are worse in the evening or at night than during the day; and symptoms that are not solely attributable to other medical or behavioral conditions.^[11]^

This study was approved by the Ethics Committee of the Istanbul Training and Research Hospital. (2023-195); the participants were informed about the examinations, and we obtained necessary consent.

### 2.1. Statistical analysis

A sample size of 100 patients per group was calculated to provide 80% power to detect the expected difference between the 2 groups, patients with or without RLS. Categorical variables are expressed as numbers and percentages (n, %). The chi-squared test was used for categorical variables. Fisher exact test was performed in cases that did not meet the chi-squared criteria. The Kolmogorov-Smirnov test was used to evaluate the normality of the distribution of continuous variables. The Student *t* test was used to compare the means of 2 independent groups for normally distributed continuous variables. Normally distributed continuous variables are shown as mean ± standard deviation.

Age, the number of chronic diseases, sarcopenia, gender, and 25 OH Vitamin-D levels, which demonstrated statistical significance between the 2 groups, were incorporated into the univariate logistic regression analysis. Subsequently, sarcopenia, gender, and 25 OH Vitamin-D levels, which remained statistically significant in the univariate analysis, were further analyzed using multivariate logistic regression.

Before multivariate logistic regression analysis, multicollinearity among predictors was assessed using variance inflation factor, and no significant multicollinearity was detected. Variables included in the regression model were selected based on statistical significance in univariate analyses and clinical relevance. In addition, the assumptions of logistic regression were evaluated prior to analysis. Sensitivity analyses were not performed, as the multivariable model results remained stable after adjustment.

The SPSS-22 statistical program was used for the analysis, and *P* <.05 was considered statistically significant.

## 3. Results

A total of 114 patients diagnosed with RLS (Group 1) and 147 patients without RLS (Group 2) were included in the study. The mean age of patients with RLS was 70.11 ± 9.56 years, whereas that of the group without RLS was 71.76 ± 9.61 years. Although there was no significant difference in age between the groups (*P* = .170), the number of female patients was significantly higher in Group 1 (*P* = .001). There were no significant differences in terms of chronic diseases, including hypertension, diabetes mellitus, coronary artery disease, osteoporosis, hypothyroidism, asthma, chronic obstructive pulmonary disease, atrial fibrillation, and benign prostatic hyperplasia (*P* = .520, *P* = .802, *P* = .699, *P* = .219, *P* = .464, *P* = .517, *P* = .297, *P* = .845, *P* = .871, respectively). Hyperlipidemia was observed in 17% of patients with RLS and 19% of patients without RLS, and this difference was not statistically significant (*P* = .610). All patients who used medication for hyperlipidemia were in the statin group. Additionally, no significant difference was found in the number of chronic diseases and medications used (*P* = .167 and *P* = .237, respectively). We found no significant difference in the laboratory values of hemoglobin A1c, ferritin, creatinine, hemoglobin, thyroid-stimulating hormone, and vitamin B12 levels (*P* = .594, *P* = .398, *P* = .138, *P* = .780, *P* = .812, *P* = .233, respectively). However, Group 1 had significantly higher vitamin-D levels (*P* = .038). The detailed analysis is presented in Table 1.

**Table 1:** Demographic data, chronic diseases and laboratory values of patients with/without RLS.

	Patients with restless leg syndrome	Patients without restless leg syndrome	*P*
Number of patients (n=)	114	147	–
Gender (female/male) (n=)	93/ 21	92/ 55	**.001**
Age*	70.11 ± 9.56	71.76 ± 9.61	.170
Number of diseases* (n=)	2.63 ± 1.42	2.34 ± 1.70	.167
Sarcopenia	62 (54%)	54 (37%)	**.005**
Hypertension	66 (62%)	38 (74%)	.520
Diabetes mellitus	28 (26%)	37 (28%)	.802
Coronary artery disease	6 (6%)	6 (5%)	.699
Osteoporosis	12 (14%)	15 (17%)	.219
Hypothyroidism	19 (18%)	19 (14%)	.464
Asthma	5 (5%)	4 (3%)	.517
Hyperlipidaemia	18 (17%)	25 (19%)	.610
Chronic obstructive pulmonary disease	4 (4%)	9 (7%)	.297
Atrial fibrillation	12 (%11)	16 (%12)	.845
Benign prostatic hyperplasia	6 (6%)	8 (6%)	.871
Number of drugs used* (n=)	3.94 ± 2.63	3.68 ± 2.98	.237
Haemoglobin A1c (%)*	6.18 ± 1.42	6.09 ± 1.20	.594
Ferritin (ng/mL)*	49.6 ± 38.6	54.1 ± 38.7	.398
Creatinine (mg/dL)*	0.86 ± 0.26	0.91 ± 0.27	.138
Haemoglobin (g/dL)*	12.81 ± 1.36	12.95 ± 1.54	.780
TSH (µIU/mL)*	2.24 ± 1.50	2.29 ± 1.61	.812
25-Hydroxy Vitamin-D (µg/L)*	29.12 ± 14.49	25.38 ± 12.34	**.038**
Vitamin B12 (pg/mL)*	354.7 ± 214.9	370.5 ± 254.9	.233

RLS = restless legs syndrome, SD = standard deviation.

* Data are shown as mean ± SD or median (interquartile intervals), statistically significant *P*-values are indicated as bold.

Sarcopenia was diagnosed in 62 (54%) patients in Group 1, whereas the incidence was 54 (37%) in Group 2, and Group 1 had a significantly higher frequency of sarcopenia (*P* = .005). There was no significant difference between the 2 groups with regard to the SARC-F score and muscle mass (*P* = .227 and *P* = .218, respectively). However, muscle strength and gait speed were significantly lower in Group 1 (*P* = .008 and *P* = .013, respectively) (Table 2). Univariate logistic regression analysis was applied to age, gender, number of chronic diseases, sarcopenia, and 25 OH vitamin-D level. Multivariate logistic regression analysis was performed on sarcopenia, gender, and 25 OH vitamin-D, which were found to be statistically significant. However, 25 OH vitamin-D was not found to be significant in the multivariate logistic regression. As a result of the multivariate logistic regression analysis, it was determined that both sarcopenia and female gender were associated with RLS [odds ratio 1.941 (95% confidence interval: 1.107–3.402, *P* = .021; odds ratio 2.733, (95% confidence interval: 1.442–5.177, *P* = .002]. Detailed analysis is presented in Table 3.

**Table 2:** Analysis of sarcopenia and related sub-parameters in patients with/without RLS.

	Patients with restless leg syndrome (n = 114)	Patients without restless leg syndrome (n = 147)	*P*
Sarcopenia	62 (54%)	54 (37%)	**.005**
SARC-F	2.52 ± 2.10	2.19 ± 2.11	.227
Grip strength (kg)	20.50 ± 6.98	23.16 ± 8.70	**.008**
Muscle mass (SMI) (kg/m^2^)	6.65 ± 0.58	6.77 ± 0.70	.218
Gait speed (m/sn)	0.72 ± 0.27	0.93 ± 0.38	**.013**

Data are shown as mean ± SD, statistically significant *P*-values are indicated as bold.

RLS = restless legs syndrome, SARC-F = strength, assistance with walking, rising from a chair, climbing stairs, and falling, SD = standard deviation, SMI = skeletal muscle index.

**Table 3:** Univariate and stepwise multivariate LR analysis of prediction of RLS.

	Univariate LR		Stepwise multivariate LR	
	Odds ratio (95% CI)	*P*	Odds ratio (95% CI)	*P*
Gender (female)	2.648 (1.483–4.726)	**.001**	2.733 (1.442–5.177)	**.002**
Sarcopenia	2.031 (1.234–3.345)	**.005**	1.941 (1.107–3.402)	**.021**
Age	0.982 (0.957–1.008)	.171	–	**–**
Number of diseases	1.121 (0.953–1318)	.168	–	**–**
25-Hydroxy vitamin-D	1.021 (1.001–1.042)	**.042**	1.015 (0.994–1.037)	.154

Statistically significant P-values are indicated as bold.

CI = confidence interval, LR = logistic regression, RLS = restless legs syndrome.

## 4. Discussion

RLS and sarcopenia are significant contributors to morbidity, adversely impacting the quality of life among the elderly population. This study has demonstrated that sarcopenia is more prevalent in individuals with RLS, and it is not uncommon for these 2 conditions to coexist. Moreover, a notable finding of this study is the increased prevalence of RLS among women, observed not only in the adult demographic but also within the elderly cohort.

Upon reviewing the existing literature, we identified a lack of studies examining the association between restless leg syndrome and primary sarcopenia. In a study conducted by Yildirim et al, published in February 2025, a cohort of 109 patients with RLS and 220 patients without the condition was analyzed. The findings indicated that sarcopenia was significantly more prevalent among patients with RLS (*P* = .047).^[19]^ A significant limitation of this study is its retrospective design and the exclusion of secondary causes of sarcopenia. Additionally, the prevalence of sarcopenia was notably higher in the group with restless leg syndrome in our study (*P* = .005). The strength of our study lies in its prospective design, as it exclusively included patients with primary sarcopenia, thereby excluding secondary causes of the condition.

Sleep-onset insomnia is a common issue in severe RLS and affects nearly all patients. Experimental studies have shown that brain iron deficiency causes hyperdopaminergic and hyperglutamatergic states, leading to dysfunction in cortico-striatal-thalamic-cortical circuits. Brain iron deficiency may lead to a hypo-adenosinergic state in the striatum, which promotes increased glutamate release and a lack of downregulation of the dopaminergic pathway, leading to nocturnal hyperarousal, fragmented sleep, and sensory discomfort.^[20]^ Daytime sleepiness is also frequently reported in individuals with RLS.^[21]^ Insufficient sleep has been associated with a decline in musculoskeletal health. Approximately 20% of genes in skeletal muscle follow a 24-hour rhythm, and clock gene mutations have shown that the molecular clock helps protect against sarcopenia. Increased circadian cortisol levels with age can activate transcription factors of the forkhead box protein O (FoxO) family, promoting muscle atrophy. Disruption of the circadian clock and inflammatory mediators can trigger muscle catabolism.^[22]^ These findings indicate that disturbances in circadian rhythms can negatively affect the oxidative and mitochondrial functions of muscles, increasing the risk of sarcopenia.^[23]^ On this basis, the sleep disturbances frequently reported in RLS may represent a plausible contributing pathway to sarcopenia-related mechanisms; however, whether this association reflects a direct causal relationship or a shared pathophysiological substrate remains to be established through longitudinal and mechanistic studies specifically designed to address this question in the RLS population.

Inflammatory mechanisms may also play a role in RLS pathogenesis, as indicated by increased plasma levels of C-reactive protein and a high neutrophil-to-lymphocyte ratio, although further research is needed to fully understand these associations.^[24]^ “Inflammageing” refers to a chronic, low-grade inflammatory state that occurs with aging. This condition is linked to a loss of skeletal muscle mass, driven by increased levels of pro-inflammatory cytokines, such as TNF-α, IL-1β, IL-6, and IFN-γ. These cytokines enhance protein breakdown and hinder protein synthesis in the muscles, leading to sarcopenia.^[25]^ Based on these data, the hypothesis has emerged that chronic low-grade inflammation may form a common pathophysiological basis in the etiopathogenesis of both sarcopenia and RLS. However, it should not be overlooked that this hypothesis represents a speculative framework based solely on indirect evidence, and that testing the biological plausibility of this proposition requires prospective, mechanistic, and interventional studies.

RLS demonstrates an upregulation of the estrogen signaling pathway, which is associated with its higher prevalence in women compared to men.^[26]^ Notably, the prevalence of RLS increases with advancing age, affecting postmenopausal women as well, despite the lack of a definitive effect from estrogen replacement therapy. This observation implies that other age-related factors may contribute to the manifestation of RLS. To enhance our understanding of RLS in females, it is advisable to conduct longitudinal studies commencing prior to a woman’s first pregnancy, with a focus on genetic, clinical, and polysomnographic variables, hormonal status, and iron metabolism.^[27]^

Multiple lines of evidence indicate a hypoxic condition in patients with RLS. Postmortem analyses have revealed elevated levels of hypoxia-inducible factor-1α (HIF-1α) in neurons of the substantia nigra, as well as an upregulation of vascular endothelial growth factor in the cortical microvessels.^[28]^ Patients with RLS demonstrate reduced oxygen pressure in their lower extremities, a condition that ameliorates with dopaminergic treatment. Furthermore, there is evidence of increased capillary tortuosity within skeletal muscles, activation of endothelial cells in the skin, and elevated levels of vascular endothelial growth factor, all of which suggest a peripheral hypoxic state in individuals with RLS.^[29]^ Although current evidence supports the existence of a peripheral hypoxic environment in patients with RLS, whether this hypoxic process directly leads to sarcopenia in this population has not been specifically investigated. In this context, peripheral hypoxia can be considered a biologically plausible mechanism that may contribute to the development of sarcopenia specific to RLS; however, prospective and mechanistic studies targeting the RLS population are needed to confirm this relationship.

Research consistently indicates that the severity of RLS symptoms is inversely related to quality of life. Both clinical and epidemiological studies have identified a significant prevalence of depressive symptoms and major depressive episodes among individuals with RLS, which are closely linked to the severity of RLS symptoms.^[30]^ Physical inactivity results in diminished muscle quality and decreased functional performance.^[31]^ In this context, the depressive symptoms associated with RLS and the accompanying sedentary lifestyle can be considered a biologically and behaviorally plausible mechanism that may contribute to a decline in muscle mass and functional capacity, and consequently to the development of sarcopenia. However, elucidating the causal aspect of this relationship requires support from longitudinal and intervention-based studies specific to the RLS population.

**Figure 1.**
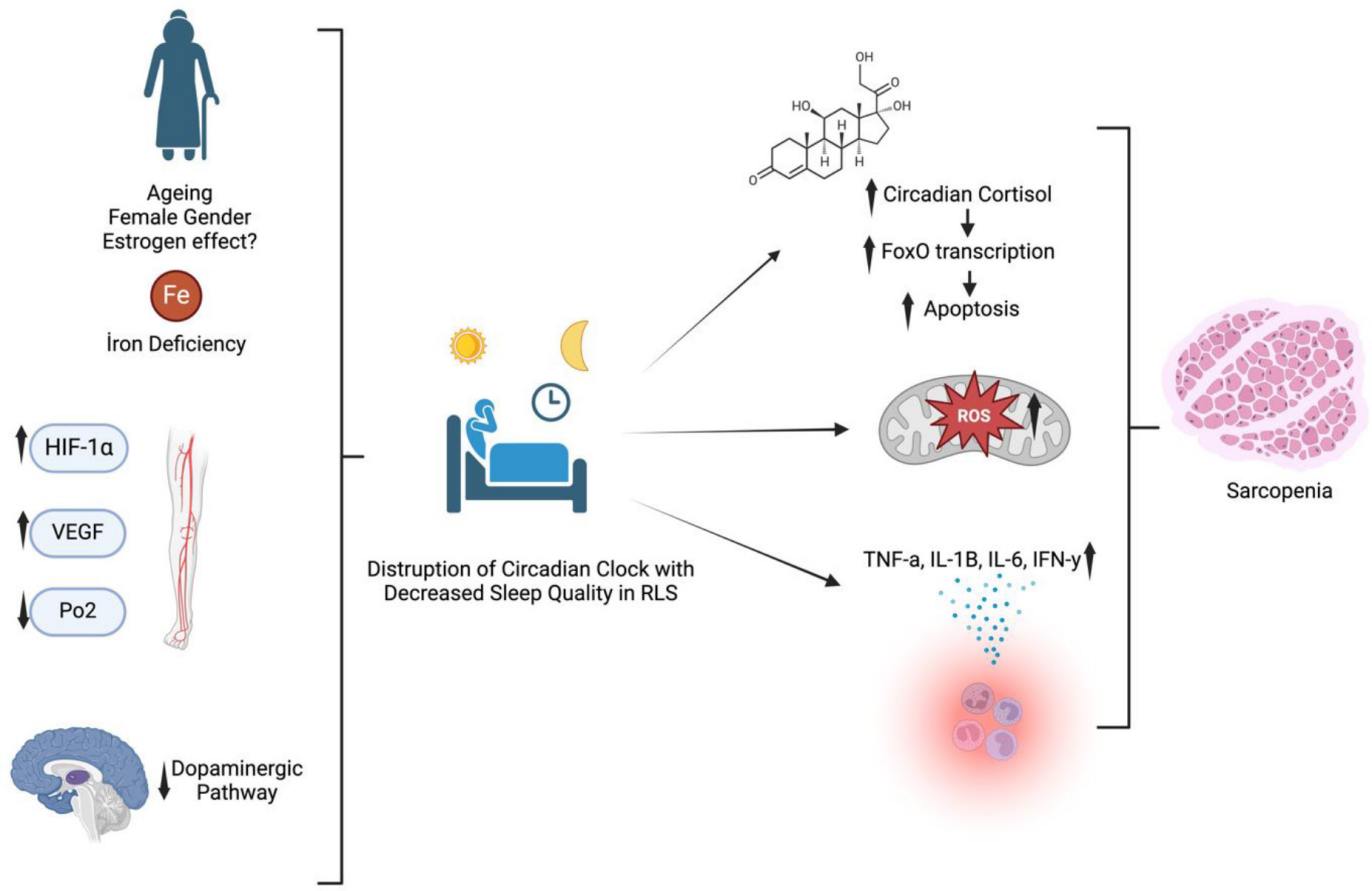
Conceptual Model Illustrating the proposed mechanistic pathways linking RLS to Sarcopenia *(Created with BioRender).* This figure illustrates 3 biologically plausible pathways through which RLS may contribute to sarcopenia development. First, RLS associated sleep disturbance may disrupt circadian rhythm integrity and impair skeletal muscle repair processes. Second, depressive symptoms and symptom related physical inactivity may adversely affect the muscle protein synthesis to breakdown balance. Third, chronic low-grade inflammation (“inflammaging”) may upregulate pro-inflammatory cytokines including TNF-α, IL-1β, IL-6, and IFN-γ thereby promoting muscle catabolism and suppressing protein synthesis. These pathways are proposed to interact in a mutually reinforcing manner; however, their causal directionality has not yet been directly established and should be interpreted within a speculative mechanistic framework. FoxO = forkhead box protein O, HIF-1α = hypoxia-inducible factor-1α, IL-1β = interleukin-1 beta, IL-6 = interleukin-6, IFN-γ = interferon gamma, RLS = restless legs syndrome, ROS, reactive oxygen species, TNF-α = tumor necrosis factor-alpha, VEGF, vascular endothelial growth factor.

**Figure 2.**
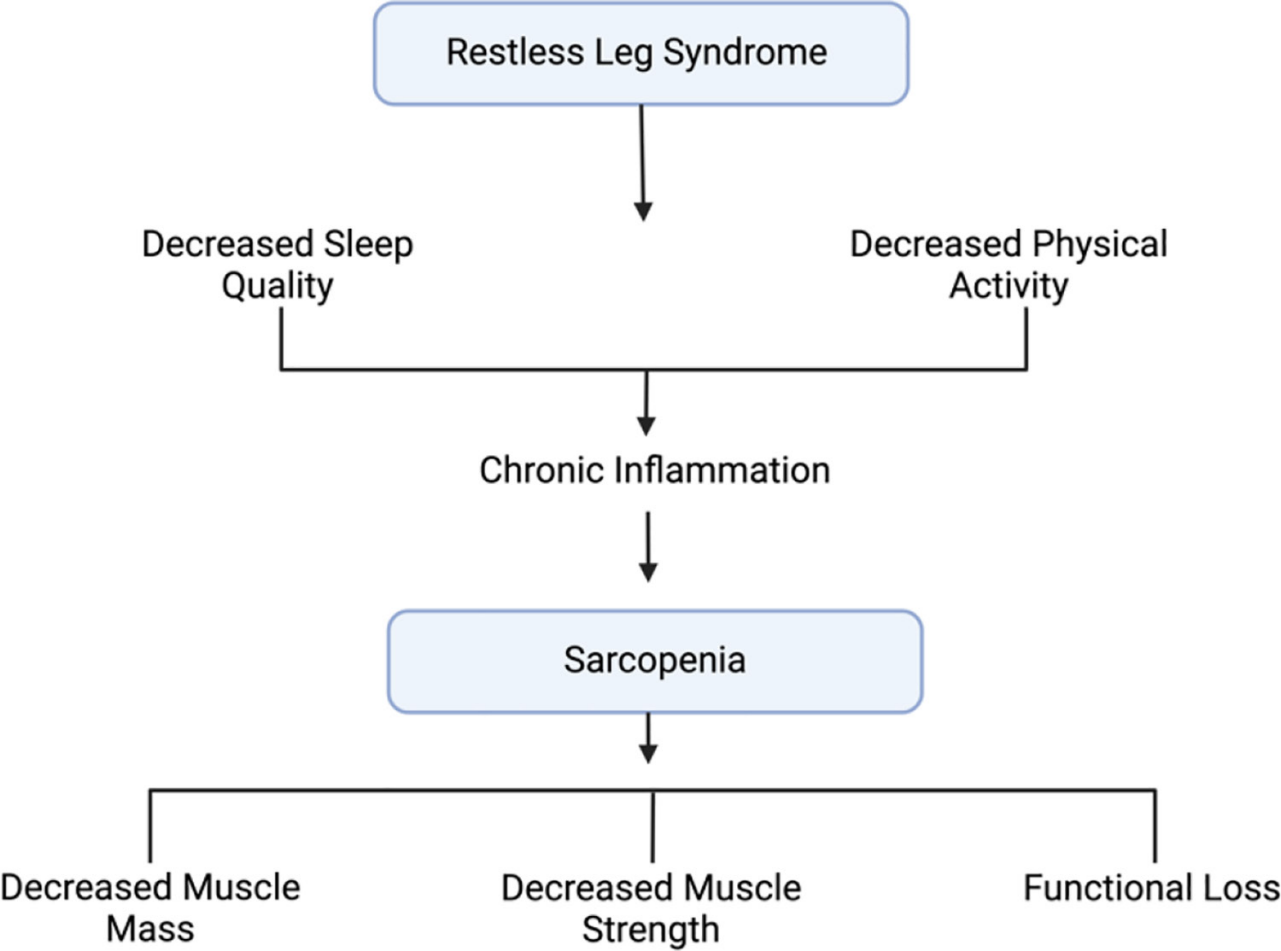
Possible association between RLS and Sarcopenia *(Created with BioRender).* This figure illustrates the potential overlapping relationship between RLS and sarcopenia, highlighting shared pathophysiological factors including dopaminergic dysfunction, chronic low-grade inflammation, iron deficiency, and sleep disturbance that may predispose individuals to both conditions concurrently. The associations depicted are based on indirect evidence and should be interpreted as a speculative mechanistic framework pending confirmation from prospective studies. RLS = restless legs syndrome.

Upon comprehensive evaluation of the extant evidence, we propose that RLS may disrupt sleep architecture, thereby shortening the processes of muscle rest and repair. Consequently, the resultant daytime sleepiness could adversely impact levels of physical activity. Moreover, the chronic low-grade inflammation associated with RLS may synergistically contribute to the loss of muscle mass and strength when combined with reduced physical activity. In this context, we posit that sleep disturbance, physical inactivity, and chronic inflammation may constitute biologically plausible and potentially mutually reinforcing mechanistic pathways through which RLS could contribute to the development of sarcopenia. However, it is imperative to emphasize that this integrated pathway model remains speculative, and the specific contributions of each mechanism within the RLS population should be elucidated through prospective and mechanistic studies that directly test this relationship. It is detailed in Figures 1 and 2.

## 5. Conclusion

Primary sarcopenia is more prevalent among patients with RLS compared to those without this condition. Consequently, it is advisable to consider screening for sarcopenia in individuals diagnosed with RLS. However, the design of our study does not permit the establishment of a direct causal relationship between these 2 conditions. It remains unclear whether RLS may have led to sarcopenia or vice versa. Future research should focus on studies designed to elucidate the causal relationship between RLS and sarcopenia.

## 6. Limitation

Our study’s cross-sectional design allowed for the identification of associations; however, it limited our ability to draw causal inferences. Additionally, the study’s limitations include its single-center nature and the use of bioelectrical impedance for assessing muscle mass. Although multivariable logistic regression was used to adjust for potential confounders, residual confounding due to unmeasured or inadequately measured variables cannot be excluded. The regression model may not have fully accounted for all clinically relevant covariates despite careful variable selection based on statistical and clinical considerations.

## Acknowledgments

The authors thank Tugce Turkoglu for helping with the data analysis for this study.

## Author contributions

**Conceptualization:** Aysegul Akkan Suzan, Seyda Bilgin, Veysel Suzan.

**Data curation:** Aysegul Akkan Suzan, Seyda Bilgin.

**Formal analysis:** Aysegul Akkan Suzan, Seyda Bilgin.

**Funding acquisition:** Aysegul Akkan Suzan, Seyda Bilgin, Veysel Suzan.

**Investigation:** Aysegul Akkan Suzan, Seyda Bilgin, Veysel Suzan.

**Methodology:** Aysegul Akkan Suzan.

**Project administration:** Aysegul Akkan Suzan, Seyda Bilgin, Veysel Suzan.

**Resources:** Aysegul Akkan Suzan, Seyda Bilgin, Veysel Suzan.

**Software:** Aysegul Akkan Suzan, Seyda Bilgin, Veysel Suzan.

**Supervision:** Aysegul Akkan Suzan, Seyda Bilgin, Veysel Suzan.

**Validation:** Aysegul Akkan Suzan, Seyda Bilgin, Veysel Suzan.

**Visualization:** Aysegul Akkan Suzan, Seyda Bilgin, Veysel Suzan.

**Writing – original draft:** Aysegul Akkan Suzan, Seyda Bilgin, Veysel Suzan.

**Writing – review & editing:** Aysegul Akkan Suzan, Seyda Bilgin, Veysel Suzan.
